# Exploration of the Dynamic Properties of Protein Complexes Predicted from Spatially Constrained Protein-Protein Interaction Networks

**DOI:** 10.1371/journal.pcbi.1003654

**Published:** 2014-05-29

**Authors:** Eric A. Yen, Aaron Tsay, Jerome Waldispuhl, Jackie Vogel

**Affiliations:** 1 Department of Biology, McGill University, Montreal, Quebec, Canada; 2 School of Computer Science, McGill University, Montreal, Quebec, Canada; Princeton University, United States of America

## Abstract

Protein complexes are not static, but rather highly dynamic with subunits that undergo 1-dimensional diffusion with respect to each other. Interactions within protein complexes are modulated through regulatory inputs that alter interactions and introduce new components and deplete existing components through exchange. While it is clear that the structure and function of any given protein complex is coupled to its dynamical properties, it remains a challenge to predict the possible conformations that complexes can adopt. Protein-fragment Complementation Assays detect physical interactions between protein pairs constrained to ≤8 nm from each other in living cells. This method has been used to build networks composed of 1000s of pair-wise interactions. Significantly, these networks contain a wealth of dynamic information, as the assay is fully reversible and the proteins are expressed in their natural context. In this study, we describe a method that extracts this valuable information in the form of predicted conformations, allowing the user to explore the conformational landscape, to search for structures that correlate with an activity state, and estimate the abundance of conformations in the living cell. The generator is based on a Markov Chain Monte Carlo simulation that uses the interaction dataset as input and is constrained by the physical resolution of the assay. We applied this method to an 18-member protein complex composed of the seven core proteins of the budding yeast Arp2/3 complex and 11 associated regulators and effector proteins. We generated 20,480 output structures and identified conformational states using principle component analysis. We interrogated the conformation landscape and found evidence of symmetry breaking, a mixture of likely active and inactive conformational states and dynamic exchange of the core protein Arc15 between core and regulatory components. Our method provides a novel tool for prediction and visualization of the hidden dynamics within protein interaction networks.

## Author Summary

Cells are complex dynamic systems, and a central challenge in modern cell biology is to capture information about interactions between the molecules underlying cellular processes. Proteins rarely act alone; more often they form functional partnerships that can specify the timing and/or location of activity. These partnerships are subject to dynamic changes, and thus protein interactions within complexes undergo continuous transitions. Genetic and biochemical evidence suggest that regulation or depletion of a single protein can alter the stability and activity of an entire protein complex. Experimental approaches that detect interactions within living cells provide critical information for the dynamical system that protein complexes represent; yet complexes are often depicted as static 2-dimensional networks. We have built a system that projects *in vivo* protein interaction datasets as 3-dimensional virtual protein complexes. By using this method to approximate the diffusion of complex components, we can predict transient conformational states and estimate their abundance in living cells. Our method offers biologists a framework to correlate experimental phenotypes with predicted complex dynamics such as short or long-range effects of a single perturbation to the function of the whole ensemble.

## Introduction

The living cell is a dynamic, out of equilibrium system in which the interactions among components of multi-protein complexes undergo continuous diffusion and exchange. One of the central challenges in biology is to discover the relationship between the components contained within the complex and the function it performs. There are many experimental techniques, such as Tandem Affinity Purification Mass Spectrometry, and Yeast Two-Hybrid that seek to identify protein-protein interactions. The result usually is the addition of a novel edge to an already complicated network of protein-protein interactions. However, understanding the role of each individual protein within a protein complex has remained a challenge until recently.

New experimental techniques based on Protein-fragment Complementation Assays (*pca*) have successfully obtained structural insights from protein-interaction data [1], [2]. The DHFR-*pca* implements two complementing fragments of dihydrofolate reductase (DHFR) in a plate-based growth assay and reports interactions based on growth in the presence of the drug methotrexate. Methotrexate resistance is conferred to cells when two complementing DHFR fragments, each fused to the carboxyl terminus of a bait or prey protein, encounter each other through direct and indirect interaction of the bait and prey proteins. The formation of the functional DHFR enzyme is a reversible process that occurs when the bait and the prey proteins are within 8 nm of each other. It has been proposed in [1] that the data contained in the DHFR-*pca* dataset can be used to refine the results generated by the Integrative Modeling Platform (IMP) [3], a constraint based method for generating molecular structure. However, the IMP requires a large amount of precise structural information, e.g. X-ray crystallography or from electron micrographs, to generate models with atomic level resolution. While the *pca* dataset can be used to refine high-quality models generated by the IMP, the set of constraints obtained from *pca* can also be used to generate a coarse-grained model containing conformations that represent the spatial configuration of a single or several conformational states detected in vivo. Tools are needed for coarse-grained modeling that can be used to visualize and to interpret protein-protein interaction data, especially for protein complexes for which structural information is incomplete or unavailable. To our knowledge, this type of modeling algorithm does not exist.

In this article, we introduce MCMC-PCA^2^, a probabilistic model for inferring conformational states of protein complexes based on the network of protein-protein interactions obtained from the DHFR-*pca* dataset using a coarse-grained approximation of proteins. Our approach is based on a Markov Chain Monte Carlo (MCMC) procedure to generate an ensemble of possible conformations for a given clique obtained from the high quality DHFR-*pca* derived interactome generated by experimentally testing pair-wise interactions amongst 5756 yeast proteins [1]. Unlike Boolean network based approaches, MCMC allows a proper sampling of the structure state space. This set of conformations can be used to determine the existence of multiple conformations that fit the experimental data.

## Methods

### Algorithm design and implementation

Dynamic information is embedded within networks of protein interactions detected using *pca*. To extract this information and gain greater understanding of the dynamic behavior of protein complexes, we designed an *in silico* method for predicting diffusion and exchange of proteins resident in multi-protein complexes. Our method for model generation implements a MCMC approach to generate a representative sample of conformations based on experimental data for protein complex collected using DHFR-*pca* and the more recently developed OyCD-*pca* method [4] that has been used to detect dependencies of signaling proteins on adaptors that specify targeting [5]. The output models infer the conformational space within complexes that satisfy the protein-interaction network. We applied Principal Component Analysis (PCA) to identify different conformational states from the sampled conformations. Similar studies have previously used this approach to model DNA-DNA interactions to model chromosome conformations and identified major chromosome conformational changes between differentiated and undifferentiated cell types [6]. The MCMC-PCA^2^ software can be downloaded at http://aguada.biol.mcgill.ca/software.html.

### Input data

The MCMC-PCA^2^ algorithm requires the set of interactions amongst the proteins of interest and the shape of those proteins of interest to generate structural conformations. We generated a binary interaction matrix for each protein complex of interest derived from the DHFR-*pca* dataset and generated the radius of gyration from modeling each protein of the complex as a sphere.

### Coarse grained modeling of proteins as spheres

**Figure 1:**
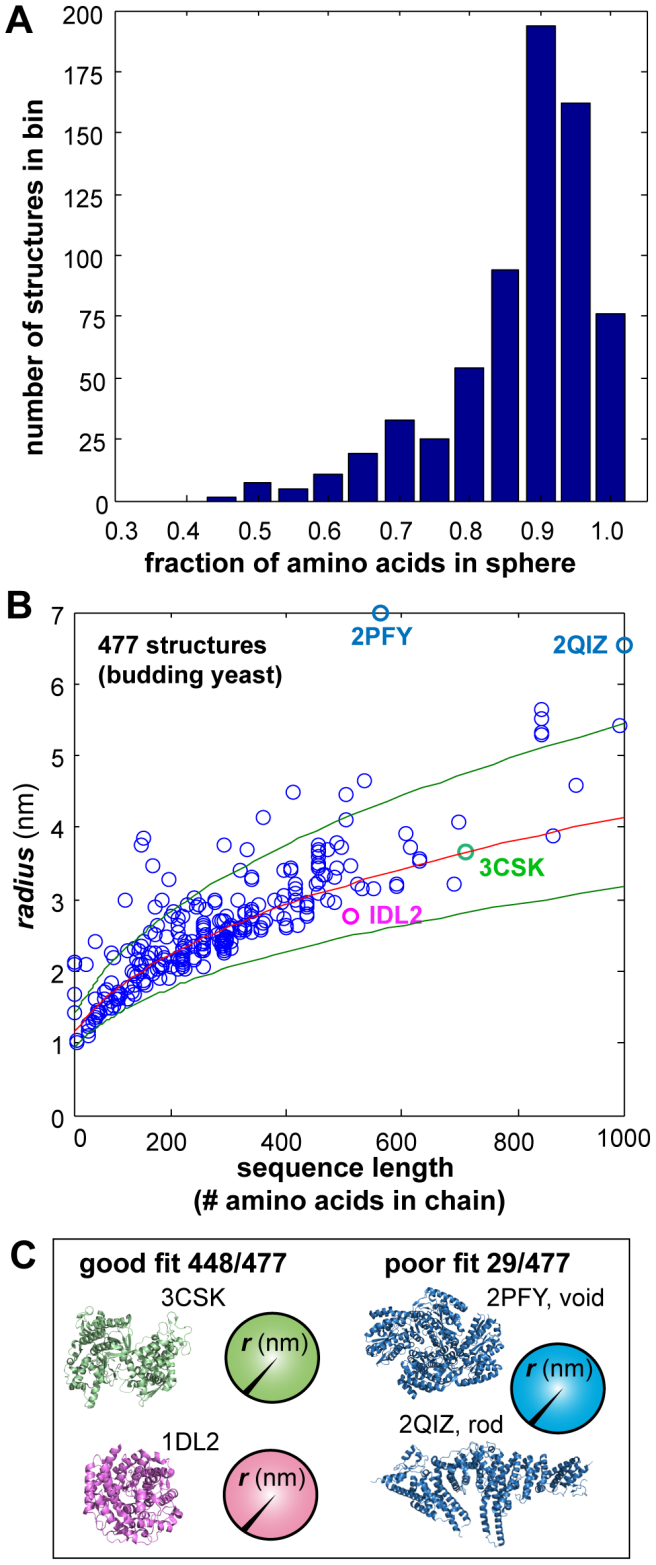
Modeling proteins as spheres. A: When structural information was available on PDB, we fit a sphere to the volume occupied by the α-carbon chain of the protein, with an initial volume of 0.5 nm and progressive increasing the volume by 0.025 nm until 100% of the α-carbons are contained within the sphere. We find that for most proteins the optimal sphere size contains 90% of α-carbons. B: The correlation between protein size (r, in nm) and chain length gives a power law (insert equation). Red: fitted line. Green: 95% confidence intervals. C: In general both globular and non-globular proteins follow the trend line. In some cases (rod shaped proteins or proteins that contain one or more clefts) protein size and chain-length are poorly correlated. (Labels: Protein Databank accession number).

To simplify the protein complex model generation, each protein within a given complex was modeled as a uniform sphere. To confirm the validity of this approach for the yeast protein dataset, we evaluated the relationship between the chain length (number of residues) and the volume occupied by the α-carbons. We evaluated all monomeric yeast proteins deposited in the Protein Data Bank (n = 477, accessed on 2012-11-28) [7]. Using the structural information for these proteins, we applied a method of “sphere-fitting”, where the objective was to find a sphere size that maximizes the number of amino acids per unit volume. This prevents overestimating sphere sizes from disordered regions protruding from the core of the protein. First, we centered a small sphere with a radius of 0.5 nm on the center of mass of each protein. Then, we expanded the sphere radius in steps of 0.025 nm, iteratively optimizing the position by maximizing the per unit volume until 100% of the α-carbons were contained within the sphere volume. We use spheres encapsulating 90% of the amino acids since this generally maximized the number of amino acids per unit volume (Figure 1A).

When structural information is unavailable, we estimated protein size from the correlation between sphere radii and amino acid sequence length from our results above. We obtained a power-law correlation (size  = 2.83 * x^0.39^), with 448 of the 477 proteins falling within the 95% confidence interval (green lines, Figure 1B), a result that strongly agrees with previous studies using this approach [8]. As expected, globular proteins lie on the trend line (Figure 1C, left). Examples of proteins for which sphere fitting to the α-carbon volume is poor include rod-shaped proteins and proteins that contain one or more large clefts (Figure 1C, right). Our predicted sizes are in good agreement with previous studies [8]. Previously, it was demonstrated that the exponent depended primarily on the hydrophobicity of the protein and not the structural features [8]. Alternatively, sphere fitting can also be applied to predicted structures such as those obtained from RaptorX [9] and Rosetta [10]. The predicted structures are expected to provide a more accurate estimation of the size of the protein, especially when the protein has homologues of known structure.

### Generating protein complexes

We define a conformation of a protein complex as a connected network such that each protein in 3-dimensional space occupies a sphere of size R_i_ at the coordinate P_i_ =  (P_x_(i), P_y_(i), P_z_(i)). The set of spheres **P** = {P(1),P(2),…,P(n)}, where n is the number of proteins in the network being simulated, constitutes a possible conformation for the protein complex. Although proteins in living cells contact each other and “overlap” (i.e., classic enzyme substrate interaction), we implemented a constraint that allows contact but prevents proteins from overlapping.

**Figure 2:**
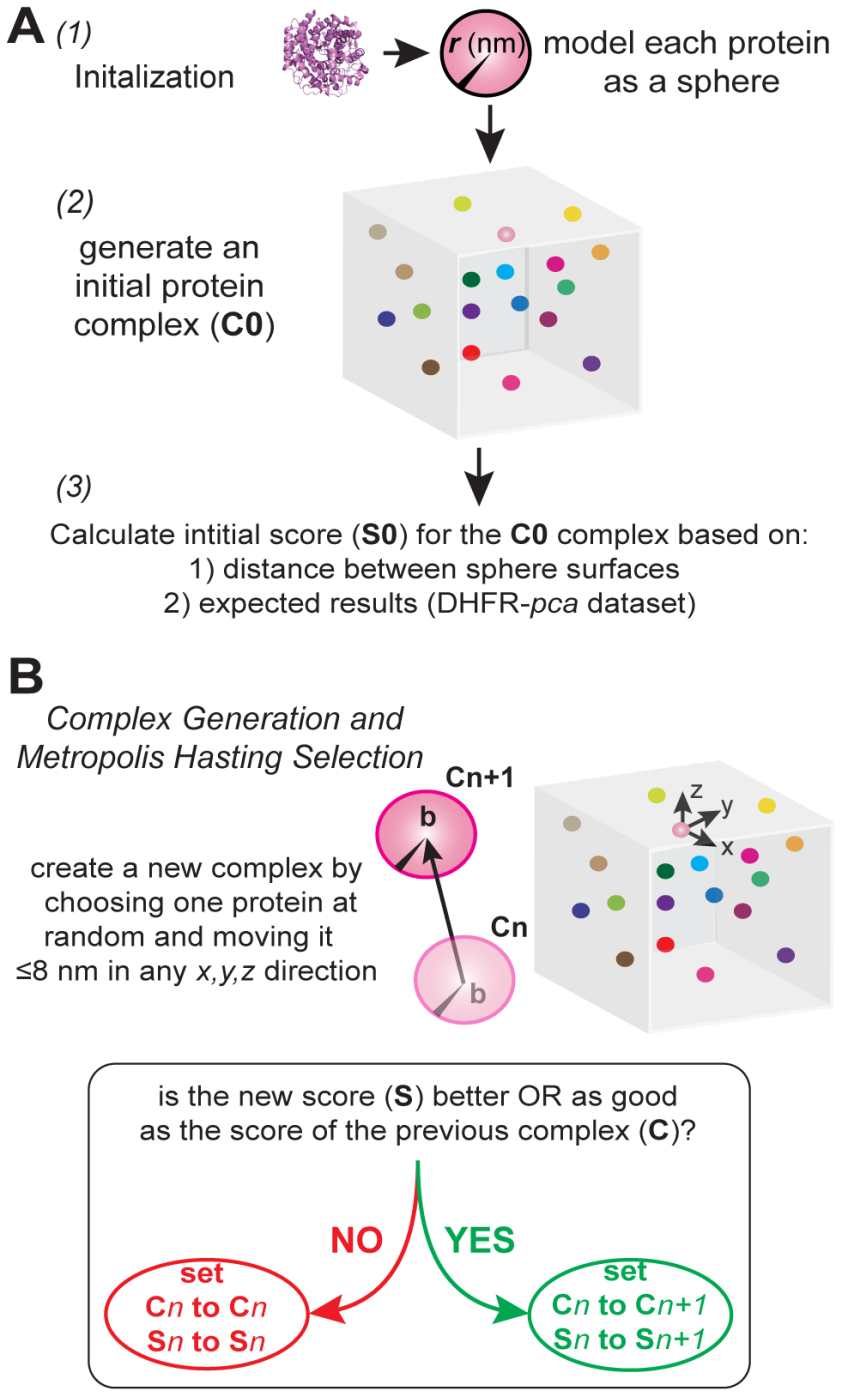
Overview of the MCMC PCA algorithm. A: Initialization of the algorithm involves three steps. The proteins are modeled as spheres (1) and randomly placed on a grid (2). This random placement constitutes as the initial conformation sampled. A score is calculated for this initial complex (3). B: At the beginning of each iteration, three protein is chosen at random (with repetition) to be translated by up to 4 nm in a random direction in x, y and z. The arrow indicates a move chosen by the algorithm. C: We accept the new complex according to the Metropolis-Hasting sampling method.

**Figure 3:**
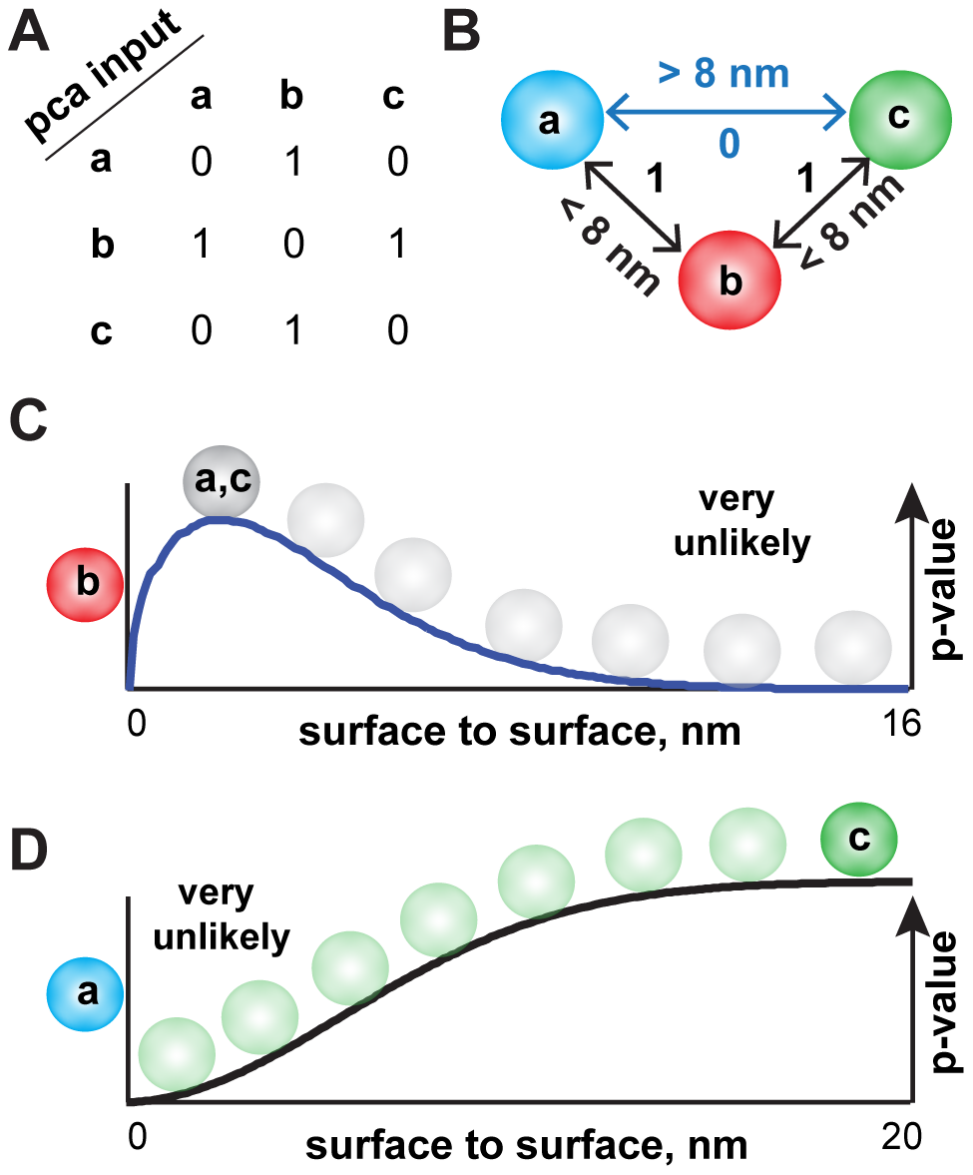
Applying MCMC-PCA^2^ to a simple toy model. A: The input data for a toy model. Let A interact with B, B with C, but that A does not interact with C. B: The toy model should satisfy these constraints, as determined by the DHFR-*pca*. C: The surface-to-surface distance between interacting pairs are modeled as Weibull distributions. This allows D: The surface-to-surface distance between non-interacting pairs is modeled as a cumulative Weibull distribution.

To generate the conformational states of a protein complex, we applied a modified Markov Chain Monte Carlo simulation (Figure 2 and supplemental text S1). During the initialization, the proteins (represented as spheres) are placed randomly on a 3D grid of 4096 nm per side (Figure 2A). We then calculate a score based on the surface-to-surface distance of each protein-pair. This score was modeled upon a knowledge-based potential (described below, Figure 3). At the beginning of each iteration, three proteins are picked randomly (with replacement) and are moved in a random direction with a maximum step size of 4 nm in x, y and z (Figure 2B). As in the initialization, we calculate a new score for the new conformation. Then, we apply Metropolis-Hasting selection between these two conformations and move on to the next iteration (Figure 2 and supplemental text S2) [11]. We say the system has sufficiently mixed after k iterations, such that the lag-1 autocorrelation of the score of the conformations sampled at k/10, 2k/10, … 10k/10, is less than 0.3. We then sample a conformation after every k/10 iterations. In order to generate a large number of possible conformations, we simultaneously ran 4,096 independent MCMC simulations generating 20,480 sampled conformations. The approach of lowering the number of iterations per simulation and increasing the total number of independent simulations takes advantage of the speed and extreme parallelization offered by general purpose computing on graphics processing units using CUDA [12].

### Modelling the knowledge-based potential

Our algorithm relies on the precise, binary nature of the *pca* method that detects well-defined, pair-wise protein-protein interactions. For all interaction pairs, a positive outcome implies that the two proteins of interest come together such that the mutant DHFR enzyme forms and can overcome the inhibition of methatrexate. The spatial constraint of the DHFR-*pca* was estimated using the RNA polymerase II crystal structure as a benchmark to reference detected interactions against the distance between carboxyl termini of protein pairs. Positive *pca* interactions were predominantly composed of protein pairs within 8 nm of each other with an upper bound of ∼ 11 nm [1].

The estimated 8 nm resolution of the DHFR-*pca* method is ∼2 fold greater than the average diameter of a folded protein [13]. Our coarse-grained approach of modeling proteins as spheres has the potential to overestimate protein size, however this nanometer imprecision is overshadowed by the resolution of the *pca* method. Moreover, the 8 nm resolution of the assay implies pairs of interacting proteins have significant diffusional space in which to assume conformational states.

The spatial constraint implicit in the interaction dataset represents a physical barrier that is overcome if an interaction is detected, and must be factored in to the knowledge-based potential. Proteins that are more than 8 nm apart are much less likely to be detected as interaction [1] and our simulation requires that we take the 8 nm resolution of the DHFR-*pca* method into consideration when modeling the surface-to-surface distance between interacting and non-interacting proteins.

As designed, the DHFR-*pca* could only detect interactions between proteins for which the C-termini are separated in distance by no more than approximately 8 nm [1]. Consequently, the expected distance between most of the interacting proteins would be less than 8 nm and very few of the detected interactions would occur at the 8 nm limit. This is a reasonable assumption when considering the true physical limitation of the *pca* assay. We also consider the flexible linker (GGGGS)_2_) between the protein of interest and the DHFR fragment is predicted by Pep-fold to be ∼1.2 nm in length when fully relaxed and able to stretch up to ∼2.4 nm [14]. Given that the DHFR protein has a radius of gyration of ∼2.2 nm, the DHFR-*pca* assay could detect binary protein-protein interactions at up to about 9.2 nm if both linkers are fully stretched. It would be rare for the linkers to be consistently stretched to full length during the detection of an interaction. Based on this, the probability of detecting an interaction should rapidly decrease as the distance between the proteins increase since the stretching of the linker would be thermodynamically unfavorable. This creates a bias in the distribution of the distance at which a protein-protein interaction is detected as a result of the linker not completely stretching. Therefore, in our simulations, we model the surface-to-surface distance between two proteins as a Weibull distribution.

The Weibull distribution is characterized by two parameters: scale (*λ*) and shape (*k*). The scale para­meter was set to 4 nm (half of the DHFR-*pca* resolution[1]) because this produced a distribution with most of the weight lying within the range for a detectable interaction, while allowing for false positives. The shape parameter was set to 
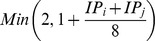
where IP_i_ is the number of interactions for protein *i*. We defined k as a dynamic parameter to prevent proteins from centering around “hub” proteins. This promotes the exchange of interaction partners to increase the conformational landscape explored. Without setting k as dynamic parameter, proteins that interact with a hub would cluster around it and create unnatural conformations.

In order to illustrate our concept, we have created a simple toy model describing interactions among 3 proteins: A, B and C (Figure 3). We assume that the experimental DHFR-*pca* data shows that protein A interacts with B, B interacts with C and A does not interact with C (Figure 3A). One possible conformation is shown in Figure 3B with the constraints that are required to be satisfied (Figure 3C,D). A more detailed example is illustrated in Figure S1.

A non-interacting pair proved difficult to model because we are modeling the absence of an interaction as information. As the distance between two non-interacting proteins increases, we would expect the “probability” of non-interaction to increase to 1 because there exist a critical distance where two *pca* fragments will no longer be able to complement each other. This idea is well characterized by a cumulative distribution function. We model non-interacting pairs by implementing the potential function as a cumulative Weibull distribution function (λ = 8, *k* = 2; Figure S1). The cumulative distribution function equally weighs protein-pairs with a distance greater than 20 nm. We extended the distance between proteins from 8 to 20 nm, making the knowledge-based potential less stringent so as to accommodate for possible false negatives in the DHFR-*pca* dataset. This approach was critical to the simulation of the diffusional space that a given protein can potentially occupy, and to the identification of conformational states that can be extracted from the simulations.

### Prediction of metastable conformations

To probe for conformational states within the ensemble of 20,480 conformations generated by the MCMC simulation, we first minimized misalignment between conformations. To remove translational and symmetrical misalignment, we created a coordinate system based on four proteins, (0,x,y,z) from our input dataset. All conformations are first translated such that the center of mass (COM) of protein 0 is at the zero co-ordinate. Next, each conformation is rotated such that the COM protein X is on the positive x-axis then rotated around the x-axis such that the COM of protein Y has no z-value and its y-value is positive. Based on the COM of protein X and Y, the positive z-direction is defined such that the vectors defined from the origin to the COM of protein X and Y are a right-handed coordinate system. Based on this new coordinate system, we verify if protein Z is on the positive z-axis. If it is not, we use the mirror image around the X-Y plane such that it becomes on the positive z-axis.

To minimize rotational misalignment, we take a conformation *i* and project it by applying Kabsch's algorithm [11]-[13] onto the conformation *j* that is most similar to it. We define similarity between conformations based on the root mean squared distance (RMSD) between the projected conformation *i* and conformation *j*. The conformation *j* that is most similar to conformation *i* has the lowest RMSD. Since the position of each conformation being projected will change upon each iteration, we reapply the Kabsch algorithm until the sum of the positional difference before and after running the algorithm is less than 1.

Next we applied PCA on the Z-score transformed COM, where the distribution of the COM is transformed into a distribution of Z-scores. The Z-score transform is a necessary step because nodes with many edges are expected to diffuse less than nodes with few edges; the latter are expected to occupy a larger diffusional space in comparison. Therefore, without this Z-score transformation, it is expected that the first few principal components would correspond to proteins that moved the most. However, the goal is to identify a distinct change in the spatial occupation of a few key proteins, such as regulators or linchpin proteins that lead to the formation of distinct clusters. The Z-score transformation allows for such spatial changes to be identified. The ensemble of all protein complexes was then projected on to the two first principal components allowing the de-convolution of clusters representing dominant conformations.

## Results and Discussion

### Case study: The 18-member Arp2/3 complex

The composition and dynamic properties of multi-protein complexes can be studied experimentally using many techniques, yet the DHFR-*pca* method in particular reveals the modular and interconnected nature of protein interaction networks (PINs). As the number of components in a PIN increases, the need for *in silico* modeling and visualization of complex dynamics and diffusional space also increases. Detection of interaction pairs using DHFR-*pca* enabled the construction of a large-scale protein interaction network that contains a number of sub-networks, each containing 10s of proteins.

An example of a multi-protein complex is the evolutionarily conserved Arp2/3 complex, which is one of a number of protein complexes that contribute to the formation of actin filaments by decreasing the energy barrier required for nucleation [15]. The Arp2/3 complex is composed of seven proteins that together promote the nucleation of new actin filaments off of existing filaments, resulting in branched filaments that are required for both cell motility and endocytosis [16]–[18]. Mammalian Arp2/3 components and their respective yeast orthologs are shown in Table 1.

**Table 1:** Comparison of structural and *pca* binary interactions in the Arp2/3 complex ([37], [38]).

Human	*S.c.* Homolog	predicted edges in structure^1^	*pca* edges with core proteins^2^	*pca* edges with core+11 adapters^2^
Arp2	Arp2	5	6	8
Arp3, Arp3-β	Arp3	5	-	-
p41-Arc, Sop2h	Arc40	3	5	12
p34-Arc	Arc35	6	5	8
p21-Arc	Arc18	4	5	8
p20-ARC	Arc19	4	5	8
p16-Arc	Arc15	5	1	5

1 1K8K. Note that the last crystallizable residue was used, which may not represent the actual position of the C-terminal as the last residue was not present in the structure. ^2^From Tassarov et al. 2008.

The spatial scale of the Arp2/3 complex is small enough that all proteins pairings should be detected by the DHFR-*pca*, as the maximum distance between carboxyl-termini is <10 nm. The distance between pairs of carboxyl termini was calculated from the PDB structure 1K8K using UCSF Chimera [19]. With the exception of Arc15 (a single core interaction with Arp2), the number of edges between the seven core proteins detected by the DHFR-*pca* is in agreement with the number of predicted interactions between proteins with carboxyl termini < 8 nm apart, as well as three interactions between pairs >8 nm apart; Arp2-Arc19 (8.49 nm), Arc40-Arc18 (9.14 nm) and Arc40-Arc19 (9.21 nm; Table 1).

**Figure 4:**
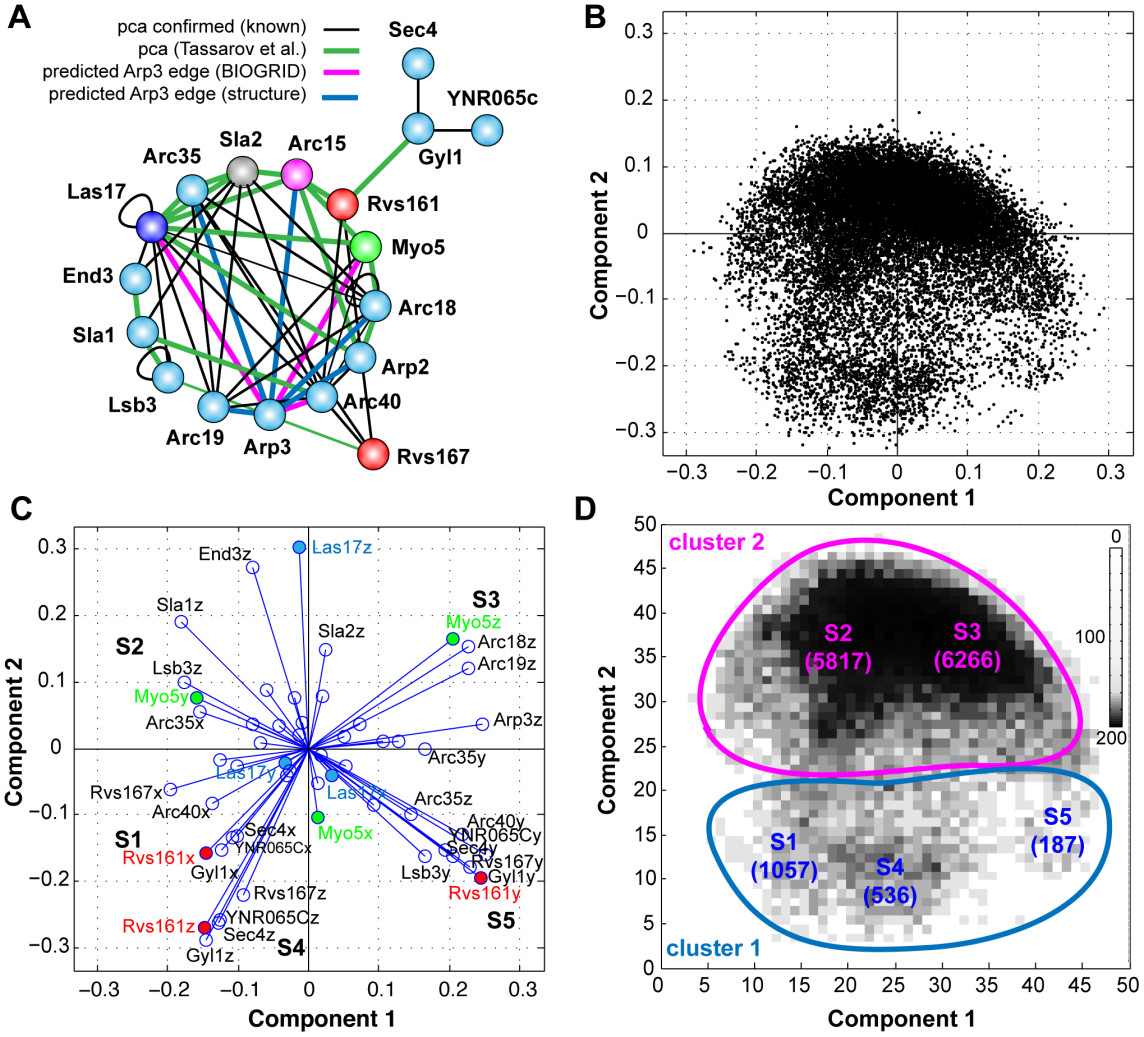
A: The experimental DHFR-*pca* interaction network. Arp3 was not included in the Tassarov *et al.* analysis and DHFR-*pca* edges are not available. Edges for Arp3 are based in predictions from the Arp2/3 structure (blue) and protein interaction data from BIOGRID (pink). B: Principal Component Analysis (PCA) was performed on the set of all conformations to project each conformation on to the two first principal components. Two clusters are formed: major (top) and minor (bottom). C: Biplot for the PCA. The biplot creates a visual representation of the contribution of each variable to the first two principal components. The length and direction of each line associated to each variable indicates its importance in the separation of the data. The PCA landscape indicates the presence of 5 different sub-regions. D: PCA scores were grouped in to a 2D histogram with sub-regions indicated. In parenthesis: number of conformations in sub-region.

The extended Arp2/3 DHFR-*pca* PIN contains 18 proteins, 11 of which are accessory proteins that are connected to one or more of the proteins that comprise the seven-member core Arp2/3 complex [1]. Sla1, Sla2, Las17, Rvs161/Rvs167 and the type I myosin Myo5 are well-characterized regulators of the actin nucleation and membrane scission functions of the complex (Table 2) [18], [20]–[22]. Las17 is the budding yeast homologue of the mammalian Arp2/3 activating protein WASp [20]. The DHFR-*pca*-derived complex is both complete and appropriate as a test bed for our analysis. One exception is Arp3, which was not tested because the DHFR-tag produces a non-functional Arp3 protein. Then, to predict interaction edges between Arp3 and members of the extended complex, we used structural information from the 1K8K structure as well as interaction data in BioGrid information. The full interaction matrix for all 18 proteins is shown in Figure 4A and Supplementary dataset S1. When proteomic data are unavailable from other experimental sources, predicted protein-protein interactions, such as those obtained from Coev2Net [23] can be used as input data to the algorithm.

**Table 2:** Accessory proteins identified in an extended Arp2/3 complex by Tassarov**.**

protein	*S.c.* homolog	function
WASp	Las17	Actin nucleator
Myosin I	Myo5	Type I myosin associated with branched actin filaments
	Sla1	Adaptor protein that links actin to clathrin in endocytosis
Hip1/R	Sla2	Adaptor protein that links actin to clathrin in endocytosis
	Lsb3	
	End3	
Amphiphysin	Rvs161	Required for membrane scission during endocytosis
Amphiphysin	Rvs167	Required for membrane scission during endocytosis
	YNR065C	Uncharacterized
	Gyl1	GAP with a role in exocytosis
	Sec4	Involved in exocytosis

### Prediction of metastable states of the 18-component complex

In order to investigate the possible conformational states embedded within the extended Arp2/3, we generated an ensemble of 20,480 different conformations. We align the conformations using Arc15, Arp2, Arc40 and Sla2 and minimized their rotational and symmetrical misalignment as described in the previous section. We then performed principal component analysis (PCA) on the aligned conformations. These aligned conformations were then projected onto the first two principal components (Figure 4B). PCA allows us to visualize the distribution of predicted conformations of the Arp2/3 complex.

The PCA analysis revealed interleaved yet distinct clusters, and indicates a continuous transition from one conformation to another with at least one major conformational state and one minor conformational state (Figure 4B). We investigated this possibility by creating a biplot, which creates a visual representation of the contribution of each variable (the x, y, z components of a protein) to the first two principal components (Figure 4C). Each line represents the relative contribution of that variable to the first two principal components and was used to identify the proteins that contribute to five different sub-regions of the PCA landscape (Figure 4D). Interestingly, many of the proteins that contribute to the formation of these clusters are regulatory proteins that include Myo5 and Las17 (activators of the complex) and Rvs161/167 (effector proteins). Surprisingly, Sla1, an inhibitor of Las17, is an important factor contributing to the formation of sub-region 2 only, but seems to have little influence on the core Arp2/3 complex, suggesting that cluster 2 represents a large population of extended complex conformations (Figure 4D). In other sub-regions, the regulators and effectors have a stronger influence on the core complex and most strongly in sub-region 3 where Myo5, a motor protein, is thought to activate the Arp2/3 complex and couple its activity to the curvature of the plasma membrane during endocytosis [24]. This biplot would suggest that there is a spatial coordination between the core Arp2/3 complex and its regulators and effectors.

### Minor and major clusters suggest distinct branching and scission conformations

**Figure 5:**
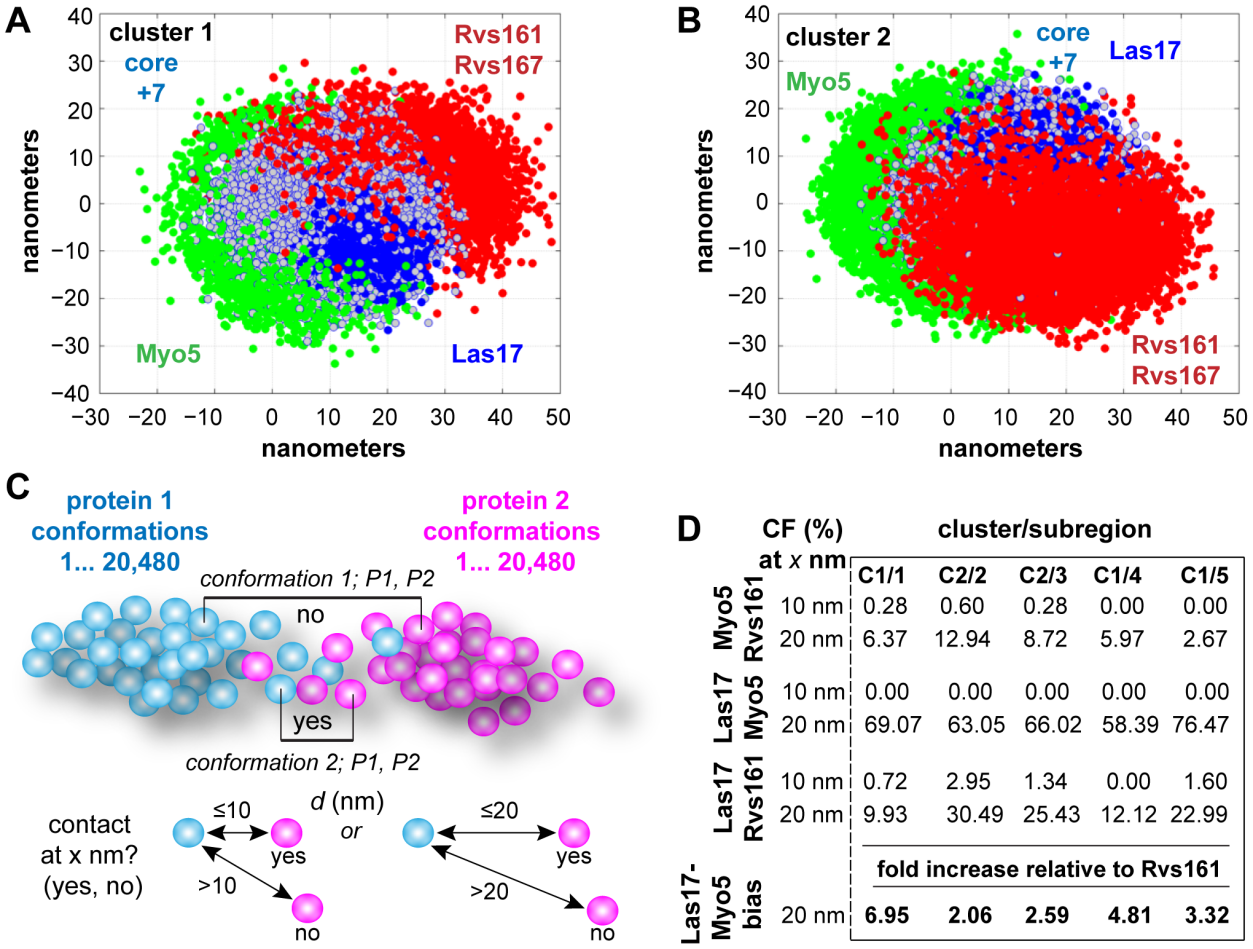
Visual representation of 3-dimensional complex conformations within cluster 1 (A) and cluster 2 (B). Distributions of Myo5, Las17 and Rvs161/7 show the most striking conformational changes across the two clusters. Green: Myo5, Dark blue: Las17, Red: Rvs161/167Blue-grey: Core+7; composed of the seven core proteins of the Arp2/3 complex and adaptor proteins Sla1/2, Lsb3, End3, Gly1, Sec4 and the expression product of YNR065C. Symmetry breaking within the extended complex suggests branching versus scission complexes. C: Contact frequency is used to quantitatively assess functional domains and spatial relationships amongst components, typically using a resolution of 20 nm. D: Contact frequency demonstrates symmetry breaking of Myo5 and Rvs161/167, and a Myo5 bias in the distribution of Las17.

Guided by the biplot, we next investigated the two major clusters of the PCA landscape by plotting the center of mass of each protein as a dot, which creates a visual representation of complex conformations. We highlighted proteins that the PCA analysis determined to be contributing the most to the formation of that cluster (Rvs161/167, Las17 and Myo5, Figure 5A,B). Each cluster is viewed from the same angle. Distributions of the accessory proteins Myo5 (Green), Las17 (Dark Blue) and Rvs161/167 (Red) are distinctive for clusters 1 and 2 in comparison with the distribution of the majority of Arp2/3 core components and accessory proteins (collectively labeled core+7).

Our analysis of complex conformations suggests spatial interplay between Myo5 and Las17 (activators of the Arp2/3 complex) and Rvs161/Rvs167 (required for scission during endocytosis) [25], [26]. Conformations resident in both cluster 1 and cluster 2 are characterized by an asymmetric distribution of Myo5 and Rvs161/Rvs167 that breaks the symmetry of the structure. While this asymmetry is qualitatively apparent, we calculate the contact frequency (CF) and apply it as a quantitative measurement of the separation of Myo5, Las17 and Rvs161/167 (Figure 5C,D). We define the CF between two proteins as the observed frequency for which their surface-to-surface distance is within a given distance (i.e. the resolution). CF enables us to estimate mixing between two distributions of protein positions. We performed a pair-wise measurement of contacts between pairs of proteins at two resolutions (10 nm and 20 nm). We first investigated the mixing of Myo5 and Rvs161 across all 20,480 conformations produced by the MCMC (Figure 5D), which ranged from 2.63% (sub-region 5) to 12.94% (sub-region 2). Next we investigated the mixing of Las17, which functions in actin branching and is functionally coupled to Myo5 [27]. In all sub-regions, Myo5 and Las17 mixing was greater than 50% (mean; 66.6%) while Rvs161 and Las17 mixing was <31% (mean; 20.19%). This analysis is in agreement with asymmetry observed between the two conformational states of the protein complex (clusters 1 & 2) illustrated in Figure 5A,B. We hypothesize that the actin branching (Myo5) and scission functions of the core+7 extended complex are spatially distinct. The complete CF analysis is given in Supplemental dataset S2.

### Identifying activity states through biased motion

By using MCMC simulations to generate conformation states, our conformational states represent an ensemble of conformations with similar scores, close to a local minimum. Embedded within these conformational states are conformations that are undergoing diffusion and exchange relative to the local maximum scoring state. The diffusional search space for each protein within a given conformational state is determined by a set of attractors (interacting proteins) and repressors (non-interacting proteins). Therefore, it is expected that proteins are continuously undergoing biased motion within a simulation. By determining the direction of the biased motion, we create a directed network of proteins moving in relation to one another. This information is useful for generating hypotheses regarding the dynamic outcomes of regulatory inputs to a macromolecular complex.

**Figure 6:**
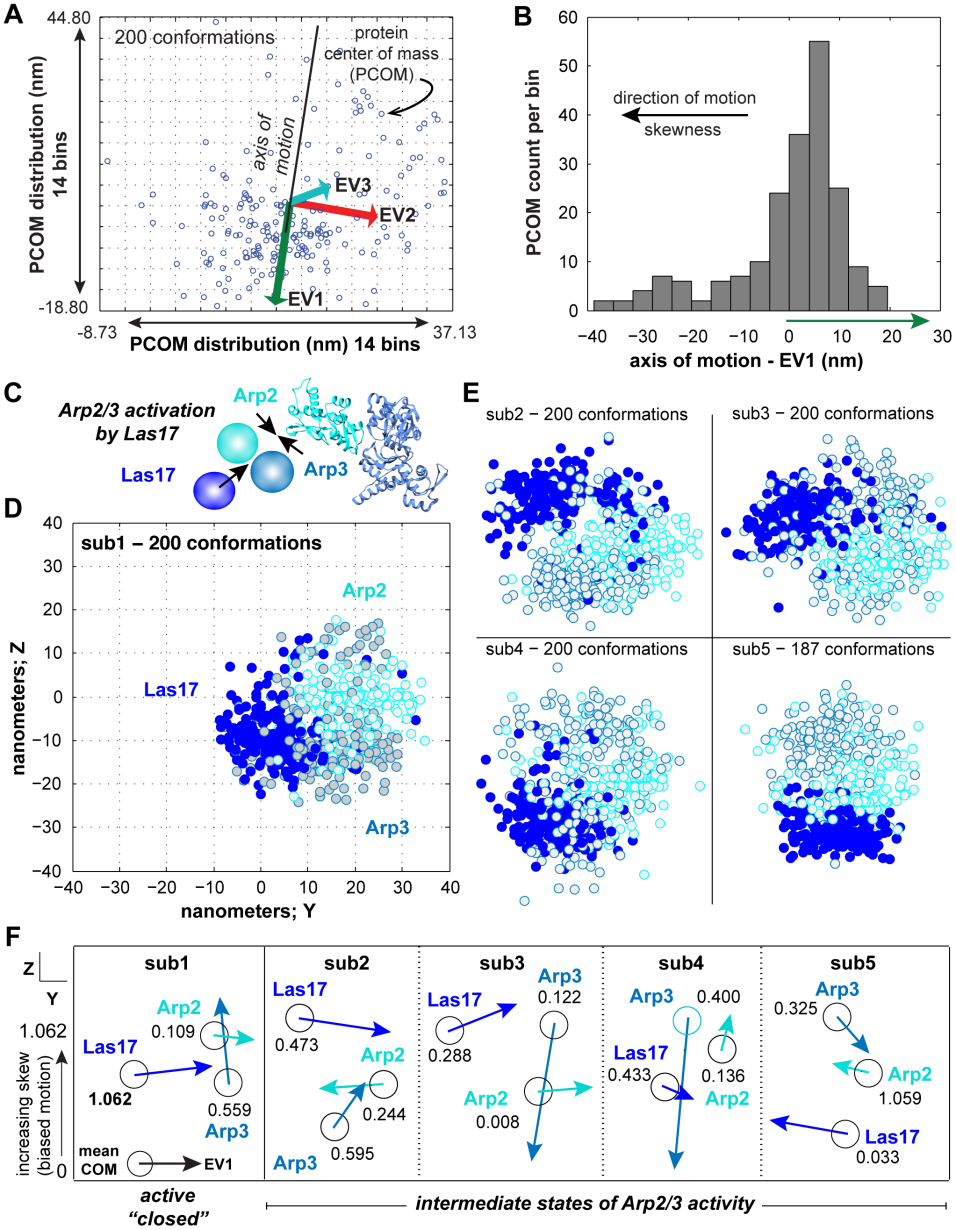
Proteins experience directed diffusion throughout a single MCMC simulation as a consequence of the set of attractors and repressors. A: The axis along the path of directed motion is expected to have the highest variance. Using the covariance matrix from the fitted Gaussian for the Protein of Interest (POI), the eigenvector (EV) with the largest absolute eigenvalue (EV1) forms the axis along which diffusion is occurring. B: Diffusion is occurring either in the direction of the EV or in the opposite direction. The long tail of the distribution determines the direction of diffusion, which can be calculated from the skew of the distribution. C: Activation of the Arp2/3 complex by Las17. Arp2 and Arp3 move towards each other, and Las17 moves towards (and binds) to Arp2 and Arp3. Sub-region 1 contains conformations that are consistent with Arp2/3 activation. D: Visualization of sub-region 1 Arp2, Arp3 and Las17 conformations. E: Visualization of Arp2, Arp3 and Las17 conformations in sub-regions 2-5. F: Directed diffusion amongst Arp2, Arp3 and Las17. Arrow length represents diffusiveness (EV1) relative to the center of mass (COM; circles) of the distribution of conformations for each protein. Numerical value is the skew, or direction of diffusion. Higher values correspond to increasing bias. In sub-region 1, Arp2 and Arp2 move towards each other and Las17 moves towards Arp2 and Arp3.

We estimated the motion of protein ensembles (i.e. ensemble of positions sampled by the MCMC for a specific protein) by fitting a Gaussian distribution to each ensemble, and quantify the bias by calculating the skewness of the distribution. First, we discretized the conformational space into 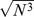 bins (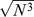 bins per axis), where N is the number of conformations, and built a 3D histogram storing frequencies of occurrence of a protein in this space. Then, we used Least-Squares minimization techniques to fit a Gaussian distribution, and used the eigenvector associated with the highest eigenvalue of the covariance matrix to determine the axis of motion (Figure 6A).

To determine the orientation of the motion, we projected the center of mass of proteins (PCOM) on this axis and estimated the asymmetry of the distribution by calculating the skewness value. The sign of this value was used to determine the orientation of the motion (Figure 6B). Then, to identify attractor(s) that may influence the movement of the protein of interest (POI), we projected the mean of each fitted Gaussian on to the main eigenvector of the POI. The POI is said to be moving towards the projected protein that is closest to the tip of the eigenvector (Figure 6B, inset).

Activation of the Arp2/3 complex by WASp/Las17 has been studied at the level of conformational changes in the seven-protein core structure. Reconstituted Arp2/3 complexes composed of budding yeast proteins revealed strong similarity to the mammalian 1K8K structure [28]. In both cases binding of WASp/Las17 occurs at a cleft between Arp2 and Arp3, and shifts the overall conformation of the complex from open (inactive) to closed (active)[28]. We hypothesized that activation of the core Arp2/3 complex would be represented by two criteria; directed diffusion of Arp2 and Arp3 towards each other, and directed diffusion of Las17 towards Arp2 and Arp3 (Figure 6C). These criteria were best satisfied by conformations in sub-region 1 of cluster 1. For sub-regions 1-4, a random sample of 200 conformations is shown (Figures 6D,E). Arp2 and Arp3 exhibit extensive mixing in sub-region 1 (Figure 6D) and Las17 diffuses towards both Arp2 and Arp3, with a skew 2-fold larger than in sub-regions 2-5 (Figure 6F). In sub-regions 2-5, Las17 diffuses away from Arp2 and Arp3 (Figure 6F), with the most “open” Arp2-Arp3 conformation in sub-region 4 (Figure 6E,F).

A third criterion for Arp2/3 activation by Las17 is its interaction with Sla1. Sla1 inhibits Las17 and restrains actin polymerization during critical steps in membrane scission [20]. Complex conformations where Las17 and Sla1 distributions do not overlap are expected to be representative of complexes active in actin polymerization and branching, and are observed in sub-region 1 (Supplementary dataset S3). Based on three criteria, we propose that sub-region 1 (1057 conformations of 12,083) contains active conformations of the Arp2/3 complex. We suggest that only a small sub-population (∼8%) of the conformations embedded in the DHFR-*pca* dataset represent complexes that are active in actin polymerization and branching.

**Figure 7:**
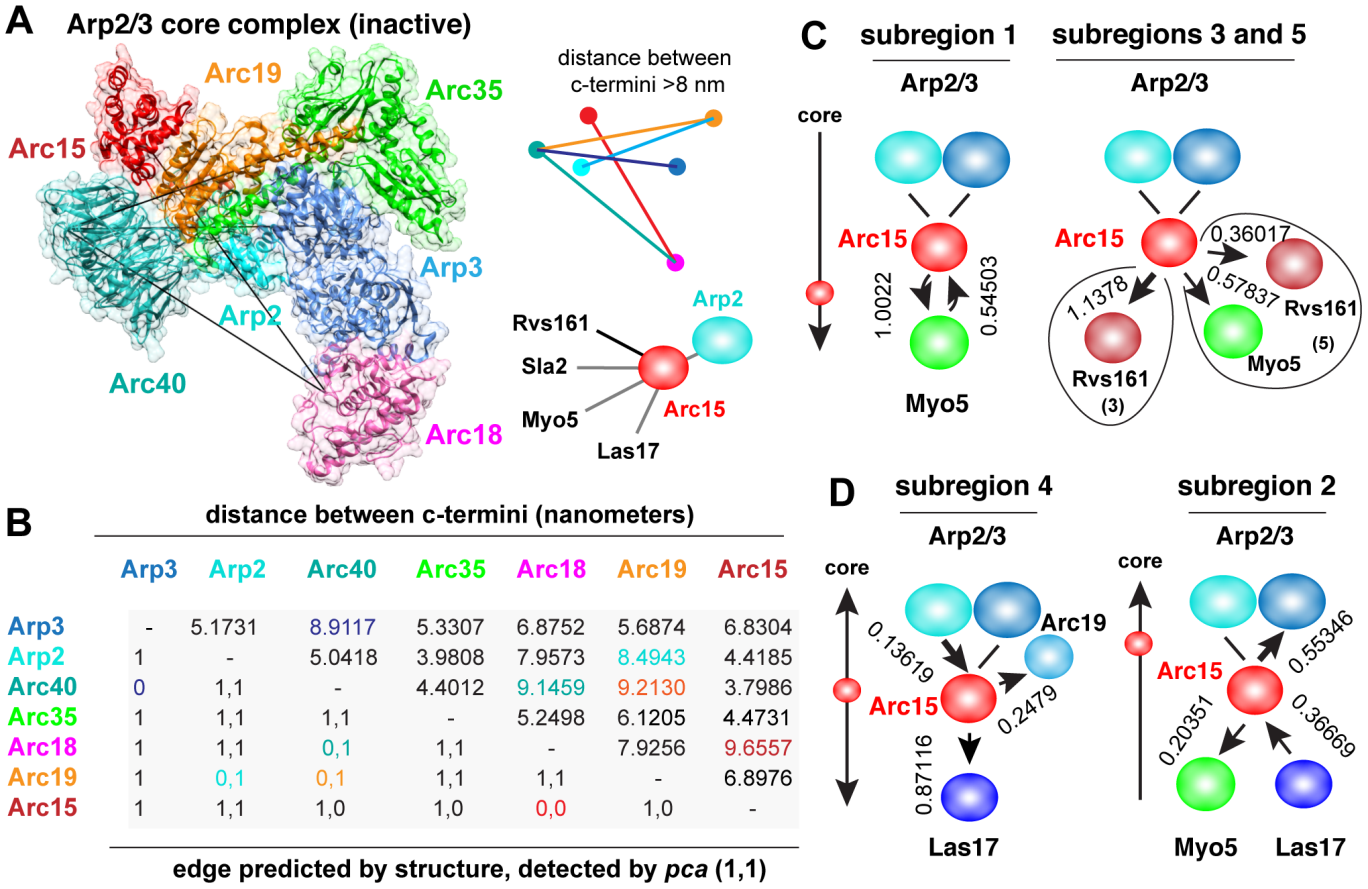
Arc15 bridges Arp2/3 core proteins and accessory proteins/regulators. A: Arc15 interacts with several accessory proteins (Las17, Myo5, Sla2 and Rvs161) and Arp2, but not other core proteins. B: Distance/edge matrix for core proteins. C: Arc15 exhibits “outward” directed diffusion towards Myo5 or Rvs161. D: Direct diffusion towards core proteins is correlated with a bias to Las17.

Arc19 and Arc35 are core components of the Arp2/3 complex that acts as scaffolds that maintain the core structure, while Arc15 contributes to the association of Arp2 and Arc40 alone [29]. Arc15 is also thought to be required for activation of the complex as a consequence of its association with Las17 [30]. Arc15 is not required for complex stability but rather links the core complex to its regulators (Figure 7A). This peripheral role of Arc15 is consistent with the DHFR-*pca* edges detected in [1], which show PPIs with Arp2 and Arc40 but not other core proteins with carboxyl termini located within 5 nm (Figures 4A and 7B). We hypothesized that these are true negatives, reflecting transient interactions between Arc15 and core proteins in living cells. Arc15's interaction with Las17, Myo5, Rvs161 and Sla2, which binds to Rvs161 and promotes membrane scission, are consistent with Arc15 serving as a tether for effector proteins that transiently couple to the Arp2/3 complex (Figure 7C,D). Our analysis of directed diffusion suggests Arc15 frequently moves towards accessory proteins such as Myo5, Rvs16. In sub-regions 1, 3 and 5, Arc15 diffuses randomly relative to Arc2 and Arc3 but towards effector proteins; Myo5 in sub-region 1, Rvs161 in sub-region 3 and both Myo5 and Rvs161 in sub-region 5 (Figure 7C). Consistent with a role as a tether, Arc15 moves towards the core protein Arc19 (sub-region 4) or Arp3 (sub-region 2), and is an attractor of Las17 (Figure 7D). Our analysis suggests the absence of DHFR-*pca* interactions between Arc15 and other core Arp2/3 proteins is a reflection of its interactions with highly diffusive accessory proteins.

In summary, our analysis suggests that MCMC-PCA^2^ is a useful method for extracting protein complex conformations representing subpopulations of protein complexes embedded within *pca-*derived datasets. We applied MCMC-PCA^2^ to the Arp2/3 complex, and analyzed the results of our simulations. As with any modeling approach, interpretations of the data are open for debate and represent hypotheses rather than conclusions. Finally, the quality/completeness of the input data influences the predicted diffusional behavior of the complex as well as the predictive power of the method. The user must remember that the model predictions for the behavior of the complex are limited to the available input data, and as with any PIN should not be assumed to be complete or accurately represent the complex as it exists in a living cell. Nonetheless, in the absence of experimental data, computational methods such as Coev2Net [23] could be used to predict missing physical constraints.

The upper bound for interaction between surfaces of interacting proteins is a physical constraint that has been experimentally determined for the DHFR-*pca* [1]. The DHFR-*pca* has been used as a molecular ruler through the implementation of several linker lengths with an extended length of 2, 4 or 12 nm (Gly_4_-Ser)_N_, where N is 1,2 or 6) [31]. This assay was able to detect the difference in spatial separation of two forms of the erythropoietin receptor (EpoR) dimer. In the absence of ligand, the distance between the EpoR subunits' COOH-termini is 7.3 nm, predicting that only DHFR-*pca* linkers with a combined extended length > 7.3 nm will consistently detect the interaction. Implementation of the (Gly_4_-Ser)_6_ linker (24 nm) resulted in the detection of ∼100% of EpoR. The (Gly_4_-Ser)_2_ linker (8 nm) detected a fraction of EpoR and ∼100% of EpoR bound to ligand [31]. The user must view the knowledge-based potential we use to model surface to surface contacts as an approximation of the true physical constraint, which should be further interrogated experimentally to test predictions of the MCMC-PCA^2^ model. It would also be theoretically possible to integrate in our framework a force field modeling the physical interactions between the proteins in the complex. Although it is out of the scope of this study, such extension has the potential to improve the accuracy of the model prediction.

MCMC-PCA^2^ can be a powerful hypothesis generation tool, as the ability to visualize and explore conformations gives the user insight into the conformations that protein complexes adopt *in vivo*, even when there is little to no structural information. Structural information is indeed important, and future development will include structural information. This method could also be applied to the analysis of PINs built using pair-wise interactions detected by other spatially defined and dynamic methods such as split-ubiqutin [32], Forster Resonance Energy Transfer [33] and Cross-linking Mass Spectrometry [34]–[36]. In the case of the Arp2/3 PIN, our findings shed light on the spatial and functional organization of a highly complex and dynamic multi-protein complex; with the prediction of the asymmetric distribution of proteins that perform actin polymerization/branching and membrane scission, a sub-population of active Arp2/3 conformations and finally unexpected insight to the dynamic association of Arc15 with the Arp2/3 complex and it's regulators. Our method extends the powerful DHFR-*pca* and OyCD-*pca* approaches for building PINs by providing a means to predict meta-states and to visualize and interrogate diffusional space among ensembles of proteins in living cells.

## Supporting Information

Dataset S1 PIN edges for the extended Arp2/3 complex. Binary interaction matrix for the 18-member extended Arp2/3 complex used in the study. A 2-dimensional representation of the matrix is shown in Figure 4A. All edges are based in interaction data with the exception of those for Arp3 are based in data from [1]. Edges for Arp3 are based on protein-protein interaction data from BIOGRID (Las17, Myo5, Arc40) or from the Arp2/3 PDB structure 1K8K (Arp2, Arc35, Arc19, Arc18, Arc15).
(XLS)

Dataset S2 Contact frequency; relates to Figure 5C,D. All values for contact frequency (%) between protein distributions for all 18 members of the extended Arp2/3 complex. CF is provided for the 5 regions shown in Figure 4D at d =  8, 10 12, 15 or 20 nm.
(XLS)

Dataset S3 Protein bias, relates to Figure 6. Results for analysis of random or biased diffusion of protein distributions in the 5 regions shown in Figure 4D.
(XLS)

Figure S1 Applying the knowledge-based potential. Toy model: Assume A interacts with B, B interacts with C, while A and C do not interact. An interaction occurs when the surface-to-surface distance between two proteins is less than 8 nm. A-H: The weighing scheme used to assign probabilities to the surface-to-surface between two interacting proteins (Weibull distribution with γ = 4, k = as shown). The number of interactions of a protein determines the mean of the distribution. As the number of interactions increases, the mean of the distribution is shifted towards the 8 nm limit. This weighting allows proteins with a large number of interactions (i.e. hubs) to displace larger sub-complexes and also sample conformations with smaller sub-complexes. I-J: For non-interacting protein pairs, we allow for a small number of experimental false negatives in our simulations by lightly penalizing protein-pairs that are within the experimental resolution of the Protein-fragment Complementation Assay.
(TIF)

Text S1 MCMC proof.
(PDF)

Text S2 Posterior sampling method (Metropolis-Hastings selection).
(PDF)
