# Correction: Exploration of the Dynamic Properties of Protein Complexes Predicted from Spatially Constrained Protein-Protein Interaction Networks

**DOI:** 10.1371/journal.pcbi.1003816

**Published:** 2014-08-11

**Authors:** The PLOS Computational Biology Staff

In the Funding Statement, the author supported by a fellowship from an NSERC training program in Cellular Dynamics of Macromolecular Complexes should be EAY, instead of AT.
