# Supplementary figures and images for: Exploration of the Dynamic Properties of Protein Complexes Predicted from Spatially Constrained Protein-Protein Interaction Networks
Source: PLoS Comput Biol. 2014 May 29;10(5):e1003654. doi: 10.1371/journal.pcbi.1003654 (PMC4038459; doi:10.1371/journal.pcbi.1003654)

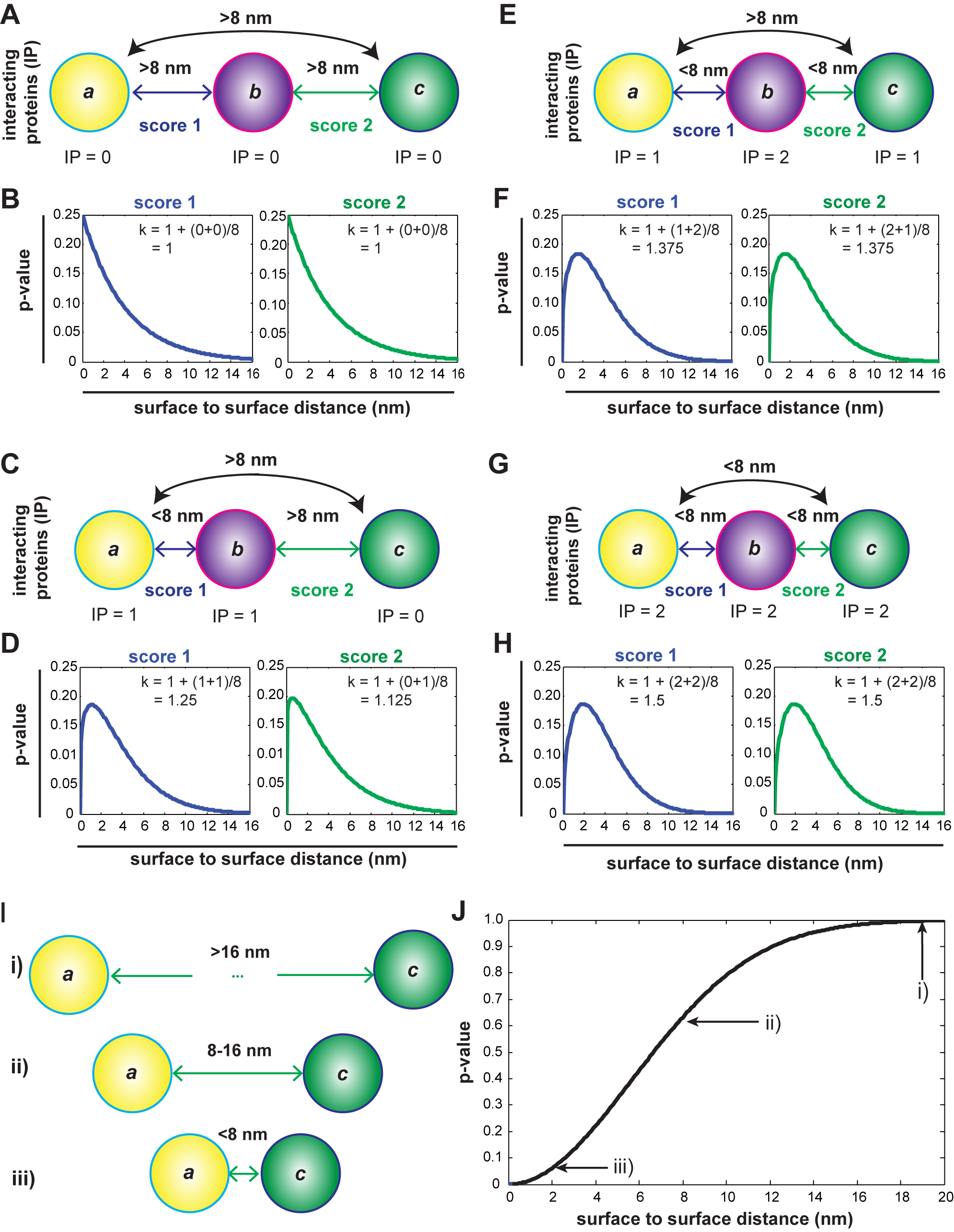

Supplement: Figure S1 — Applying the knowledge-based potential. Toy model: Assume A interacts with B, B interacts with C, while A and C do not interact. An interaction occurs when the surface-to-surface distance between two proteins is less than 8 nm. A-H: The weighing scheme used to assign probabilities to the surface-to-surface between two interacting proteins (Weibull distribution with γ = 4, k = as shown). The number of interactions of a protein determines the mean of the distribution. As the number of interactions increases, the mean of the distribution is shifted towards the 8 nm limit. This weighting allows proteins with a large number of interactions (i.e. hubs) to displace larger sub-complexes and also sample conformations with smaller sub-complexes. I-J: For non-interacting protein pairs, we allow for a small number of experimental false negatives in our simulations by lightly penalizing protein-pairs that are within the experimental resolution of the Protein-fragment Complementation Assay. (TIF) [file pcbi.1003654.s004.tif]
