# Supplementary material for: Exploration of the Dynamic Properties of Protein Complexes Predicted from Spatially Constrained Protein-Protein Interaction Networks
Source: PLoS Comput Biol. 2014 May 29;10(5):e1003654. doi: 10.1371/journal.pcbi.1003654 (PMC4038459; doi:10.1371/journal.pcbi.1003654)
Supplement: Text S1 — MCMC proof. (PDF) [file pcbi.1003654.s005.pdf]

Supplemental text S1: MCMC Proof

The observed interaction data, $\hat{I}$, defines a posterior distribution over the set of possible conformations of the protein complex: $\boldsymbol{P}[\boldsymbol{S}|\hat{\boldsymbol{I}}] = \boldsymbol{P}[\hat{\boldsymbol{I}}|\boldsymbol{S}] \cdot \boldsymbol{P}[\hat{\boldsymbol{I}}]/\boldsymbol{P}[\boldsymbol{S}]$. Since we apply a constant constraint on the state space and the probability of the observed data ($\hat{I}$) is constant with respect to S, we get $\boldsymbol{P}[\boldsymbol{S}|\hat{\boldsymbol{I}}] = \boldsymbol{\kappa} \cdot \boldsymbol{P}[\hat{\boldsymbol{I}}|\boldsymbol{S}]$ for some constant κ, and thus we have:

$$\boldsymbol{P}[\boldsymbol{S}|\hat{\boldsymbol{I}}] = \boldsymbol{\kappa} \cdot \left(\prod_{\substack{\boldsymbol{interacting} \\ \boldsymbol{i,j}}} \boldsymbol{P}[\,\hat{\boldsymbol{I}}(\boldsymbol{i},\boldsymbol{j}) |\, \boldsymbol{I}(\boldsymbol{i},\boldsymbol{j}) = \boldsymbol{f}(\boldsymbol{D_S}(\boldsymbol{i},\boldsymbol{j}), \boldsymbol{k})] \right) \cdot \left(\prod_{\substack{\boldsymbol{not-interacting} \\ \boldsymbol{i,j}}} \boldsymbol{P}[\hat{\boldsymbol{I}}(\boldsymbol{i},\boldsymbol{j}) |\, \boldsymbol{NI}(\boldsymbol{i},\boldsymbol{j}) = \boldsymbol{g}\big(\boldsymbol{D_S}(\boldsymbol{i},\boldsymbol{j})\big)] \right)$$

where *f* and *g* are the Weibull and Cumulative Weibull distribution as described in the method.
