# Supplementary material for: Exploration of the Dynamic Properties of Protein Complexes Predicted from Spatially Constrained Protein-Protein Interaction Networks
Source: PLoS Comput Biol. 2014 May 29;10(5):e1003654. doi: 10.1371/journal.pcbi.1003654 (PMC4038459; doi:10.1371/journal.pcbi.1003654)
Supplement: Text S2 — Posterior sampling method (Metropolis-Hastings selection). (PDF) [file pcbi.1003654.s006.pdf]

Supplemental text S2: Posterior sampling

The Markov Chain Monte Carlo algorithm is a method often used to sample from a posterior distribution, generating a distribution of solutions. To sample conformations, we used the Metropolis-Hasting algorithm.

From the current conformation C_n, it is randomly perturbed (see figure 2) to generate the structure C_{n+1}. We then calculate the acceptance ratio $\alpha = \frac{P(C_{n+1})}{P(C_n)}$. If $\alpha > 1$, then we accept the new proposed conformation, otherwise, we accept C_{n+1} with probability α.
